# Dexamethasone Increases the Anesthetic Success in Patients with Symptomatic Irreversible Pulpitis: A Meta-Analysis

**DOI:** 10.3390/ph15070878

**Published:** 2022-07-16

**Authors:** Lorenzo Franco-de la Torre, Eduardo Gómez-Sánchez, Nicolás Addiel Serafín-Higuera, Ángel Josabad Alonso-Castro, Sandra López-Verdín, Nelly Molina-Frechero, Vinicio Granados-Soto, Mario Alberto Isiordia-Espinoza

**Affiliations:** 1 Instituto de Investigación en Ciencias Médicas, Departamento de Clínicas, División de Ciencias Biomédicas, Centro Universitario de los Altos, Universidad de Guadalajara, Tepatitlán de Morelos 47620, Jalisco, Mexico; lorfran8888@hotmail.com; 2 Departamento de Ciencias Fisiológicas, División de Disciplinas Básicas para la Salud, Centro Universitario de Ciencias de la Salud, Universidad de Guadalajara, Guadalajara 44340, Jalisco, Mexico; eduardo.gsanchez@academicos.udg.mx; 3 Facultad de Odontología, Universidad Autónoma de Baja California, Mexicali 21040, Baja California, Mexico; nicolas_addiel@yahoo.com.mx; 4 Departamento de Farmacia, División de Ciencias Naturales y Exactas, Universidad de Guanajuato, Guanajuato 36040, Jalisco, Mexico; angeljosabad@hotmail.com; 5 Instituto de Investigación en Odontología, Centro Universitario de Ciencias de la Salud, Universidad de Guadalajara, Guadalajara 44340, Jalisco, Mexico; patologiabucal@live.com.mx; 6 Departamento de Salud, Laboratorio de Cariología y Medicina Oral, Universidad Autónoma Metropolitana-Xochimilco, Coyoacán 04960, Ciudad de México, Mexico; nmolina@correo.xoc.uam.mx; 7 Neurobiology of Pain Laboratory, Departamento de Farmacobiología, Cinvestav, South Campus, Mexico City 14330, Mexico; vinicio_granadossoto@hotmail.com

**Keywords:** dexamethasone, symptomatic irreversible pulpitis, anesthetic success, pain intensity

## Abstract

Inferior alveolar nerve block (IANB) has a high failure rate in subjects with symptomatic irreversible pulpitis (SIP). It has been suggested that drugs with anti-inflammatory activity could improve the efficacy of the anesthetic used for IANB. The aim of this study was to assess the effect of dexamethasone on the success of dental anesthesia in patients with SIP. An information search was performed using PubMed and Google Scholar. The risk of bias of the included studies was evaluated with the Cochrane Collaboration’s risk-of-bias tool. The anesthetic success rate, pain intensity (VAS), and adverse effects were extracted. Data were analyzed using the Mantel–Haenszel test and odds ratio or the inverse variance and standardized mean difference. Dexamethasone increased the anesthetic success in comparison with placebo (*n* = 502; *p* < 0.001; OR = 2.59; 95% CIs: 1.46 to 4.59). Moreover, patients who were given dexamethasone had lower pain scores at 6 h (*n* = 302; *p* < 0.001; MD= −1.43; 95% CIs: −2.28 to −0.58), 12 h (*n* = 302; *p* < 0.0001; MD = −1.65; 95% CIs: −2.39 to −0.92), and 24 h (*n* = 302; *p* < 0.0008; MD = −1.27; 95% CIs: −2.01 to −0.53) when compared with placebo. In conclusion, the systemic administration of dexamethasone increases the anesthetic success rate and improves pain management in patients with SIP.

## 1. Introduction

In the field of endodontics, symptomatic irreversible pulpitis (SIP) is a dental emergency with moderate-to-severe pain upon thermal stimulus [1,2,3,4]. In this regard, approximately 80% of patients with SIP present dental anesthesia failures after a root canal treatment [1,3,4]. The reasons that this condition makes dental anesthesia difficult are not fully known [4]. Nevertheless, the possible causes of the anesthetic failure in patients with SIP are related to the inflammatory processes—altered Na^+^ channel expression in atypical sites when compared to normal pulps [5], altered membrane potentials, and decreased excitability thresholds [6]—of the pulp [4]. 

Accordingly, various drugs that interfere with or reduce inflammation and/or pain—such as the supplementary administration of local anesthetics [3], as well as the systemic administration of nonsteroidal anti-inflammatory drugs (NSAIDs) [3], corticosteroids [7], and opioid analgesics [8,9,10,11]—have been used to relieve pain and reduce inflammation of the pulp tissue, and thus achieve the most adequate dental anesthesia during endodontic treatment [3,7,8,9,10,11]. 

Some clinical assays have been carried out to determine the effect of dexamethasone on the success of dental anesthesia in patients with SIP, and interesting results have been found [8,9,10,11,12,13,14,15,16,17,18,19,20,21,22,23,24]. The main aim of this systematic review and meta-analytical assessment was to evaluate the effects of the systemic and local administration of dexamethasone on the anesthetic success rate, pain intensity, and adverse effects in patients with SIP.

## 2. Material and Methods

### 2.1. Registration

This systematic review was developed in strict adherence to the guidelines for reporting systematic reviews and meta-analyses of studies that assess health care interventions (PRISMA) [25,26,27]. The protocol was registered in PROSPERO: CRD42021279262.

### 2.2. Focused Question

Our research team posed the following question: How effective is dexamethasone compared to a placebo or a local anesthetic in terms of anesthetic success rate and post-endodontic pain management in patients with SIP?

### 2.3. Population, Interventions, Control, and Outcomes (PICO) Approach

#### 2.3.1. Inclusion Criteria 

Population: Randomized, double-blind clinical trials.

Interventions: Administration of dexamethasone in patients with SIP.

Control: A group using placebo/a group with local anesthesia.

Outcomes: Anesthetic success rate, anesthesia depth and duration, pain intensity using the visual analog scale (VAS), pain intensity evaluated through the Heft–Parker VAS, rescue analgesic medication, and adverse effects.

These criteria were written according to the PICO recommendations [28].

#### 2.3.2. Exclusion Criteria

Clinical studies reporting a loss to follow-up of more than 20%.

High risk of bias according to the Cochrane Collaboration’s risk-of-bias tool. 

### 2.4. Information Search 

The keywords used to perform PubMed and Google Scholar searches were “dexamethasone”, “corticosteroids”, “glucocorticoids”, “symptomatic irreversible pulpitis”, “active dental pain”, and “dental pain”. At least two keywords were used for the identification of articles, i.e., “dexamethasone” AND “symptomatic irreversible pulpitis”. In addition, three filters were employed in PubMed: article type (journal article, clinical trial, clinical study, or randomized controlled trial), language (English or Spanish), and species (humans). The retrieved articles were saved for further assessment. Articles published up to 1 November 2021 were eligible. 

### 2.5. Risk of Bias Assessment

The Cochrane Collaboration’s risk-of-bias tool was employed [29,30,31]. Each of the 7 points of the tool rates the risk of bias as low-risk (green), medium-risk (yellow), or high-risk (red). Only those studies that obtained low and moderate risk-of-bias scores were considered for the qualitative and quantitative analyses [29,30,31].

### 2.6. Information Extraction 

Article ID data, design, treatments, sample size, dose, anesthetic success rate, anesthesia depth and duration, thermal or electric pulpal tests, pain intensity, rescue analgesic medication, and adverse effects were obtained.

Yavari et al. 2019 [23] reported two dexamethasone groups. Considering that both dexamethasone groups were included in the pain intensity evaluation by the VAS, the sample size of the control group was divided in half in the pooled analysis. Data of normal dexamethasone group versus the control group were designed in the meta-analysis as “a” (Yavari et al. 2019a), and long-lasting dexamethasone in comparison with the control group was included as “b” (Yavari et al. 2019b).

Two independent assessors carried out the bias assessment and data extraction. Any disagreements between them were reviewed and decided by a third evaluator, when necessary.

### 2.7. Statistical Analysis

The Review Manager 5.3 software for Windows was used for data analysis, forest plots, and funnel plots. All meta-analyses were conducted using the random-effects model. The anesthetic success was analyzed using the Mantel–Haenszel test and odds ratios (ORs). On the other hand, pain intensity was assessed with the inverse variance and standardized mean difference. The data heterogeneity was measured with the I^2^ test. Funnel plots were used to represent the publication bias of the included clinical trials. The influence of the weight of each study on the results of the meta-analysis was evaluated through a sensitivity study. A *p*-value of ≤0.05 and odds ratio or a mean difference ≥ 1 (a positive or negative value on a two-sided test) within a 95% confidence interval was considered to be a statistically significant difference [29,30,32,33,34,35]. 

## 3. Results

### 3.1. Search and Evaluation of Bias

**Figure 1:**
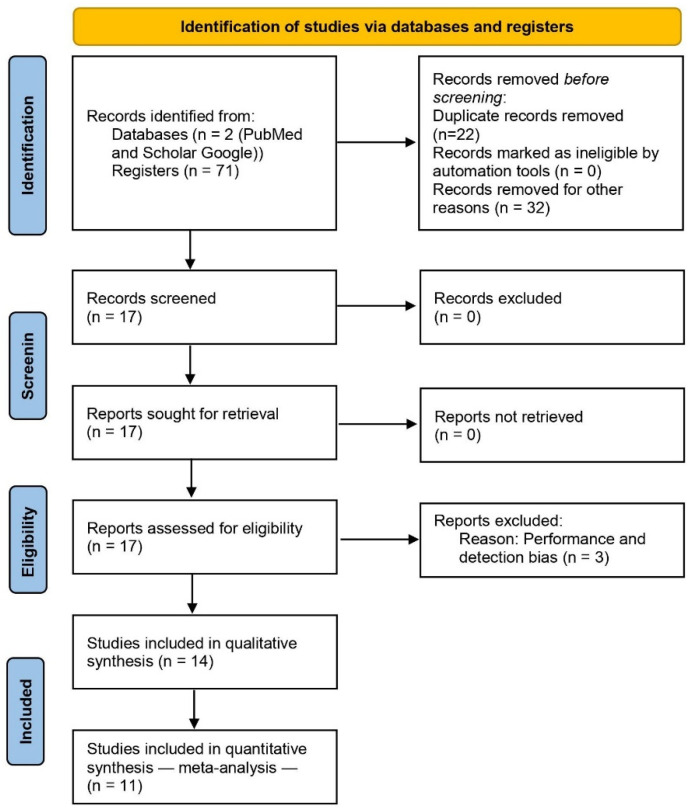
Study flowchart.

**Figure 2:**
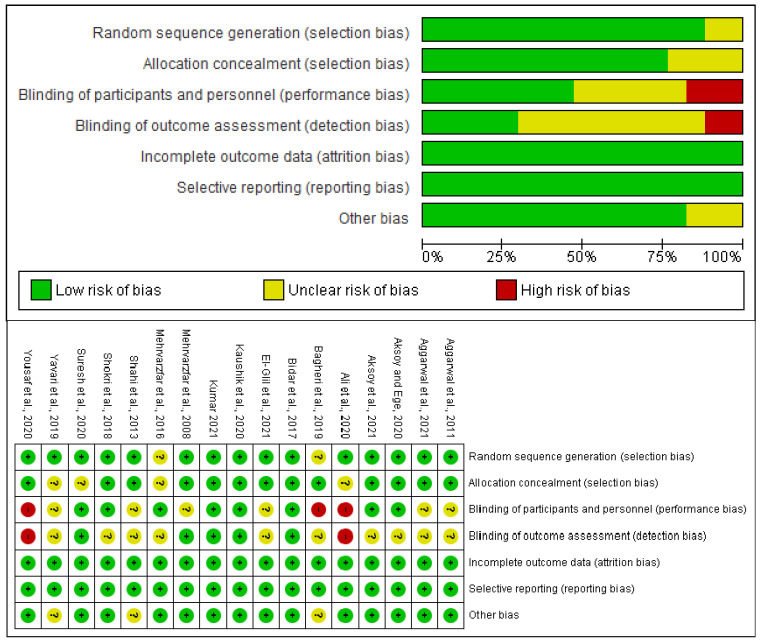
Risk-of-bias assessment.

In total, 17 clinical assays were found in both databases (Figure 1), of which 14 obtained a low or medium risk-of-bias result according to the Cochrane Collaboration’s risk-of-bias tool (Figure 2). In this way, 14 clinical trials [8,9,10,11,14,15,16,17,18,19,20,21,22,23] were used to perform the qualitative analysis, and 11 were used for the quantitative assessment [8,9,11,14,15,16,17,20,21,22,23].

### 3.2. Qualitative Analysis

The anesthetic success was evaluated across eight clinical trials that compared dexamethasone and placebo in patients with SIP [8,9,11,14,16,17,20,21]. The qualitative analysis showed that five clinical investigations presented statistical differences in favor of dexamethasone when compared to placebo [8,9,14,20,21], while three articles found no statistical difference between dexamethasone and placebo [11,16,17]. The most used anesthetic agents for IANB were lidocaine (9/14) [8,9,14,16,17,18,20,22,23], articaine (2/14) [10,11], mepivacaine (1/14) [15], while 2/14 clinical trials did not report the anesthetic agent employed [19,21]. Moreover, 4 mg was the most used dosage of dexamethasone [8,9,14,16], followed by 8 mg [10,11,15,18,19,21,22,23] and 0.5 mg [17,20]. On the other hand, nine clinical trials used the local administration of dexamethasone [8,9,10,11,15,16,18,23], while five used the oral route [14,17,19,22,23] (Table 1). 

The pain control assessment was performed with six clinical assays. All of these clinical trials found that dexamethasone was more effective than placebo in patients with SIP [10,15,18,19,22,23].

### 3.3. Quantitative Evaluation

**Figure 3:**
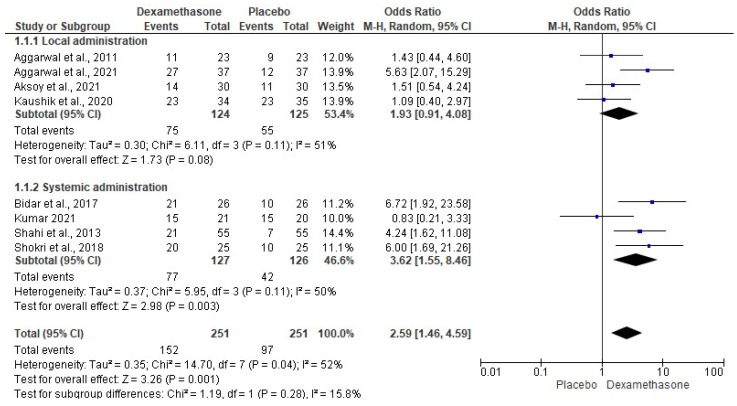
Pooled analysis of the anesthetic success rate (*p* < 0.05) [8,9,11,14,16,17,20,21].

The local assessment of the anesthetic success rate of dexamethasone and placebo was performed using four clinical studies (*n* = 249) [8,9,11,16]. Local dexamethasone and local placebo had similar anesthetic success rates (*p* < 0.08, Figure 3). Moreover, the systemic administration of dexamethasone versus systemic placebo was performed using four clinical investigations (*n* = 253) [14,17,20,21]. The systemic administration of dexamethasone showed a superior anesthetic success rate in comparison with placebo (*n* = 253, *p* < 0.003, Figure 3). Likewise, global evaluation of the anesthetic success rate was performed using data from eight clinical studies (*n* = 502). The overall anesthetic success rate of dexamethasone was 60.55%, while that of saline was 38.64% [8,9,11,14,16,17,20,21]. The pooled analysis shows a statistical difference in favor of dexamethasone when compared to placebo (*p* < 0.001, Figure 3).

Moreover, no study reported that dexamethasone improved the depth of anesthesia, whereas one dexamethasone clinical trial [11] found an increased duration of anesthesia.

**Figure 4:**
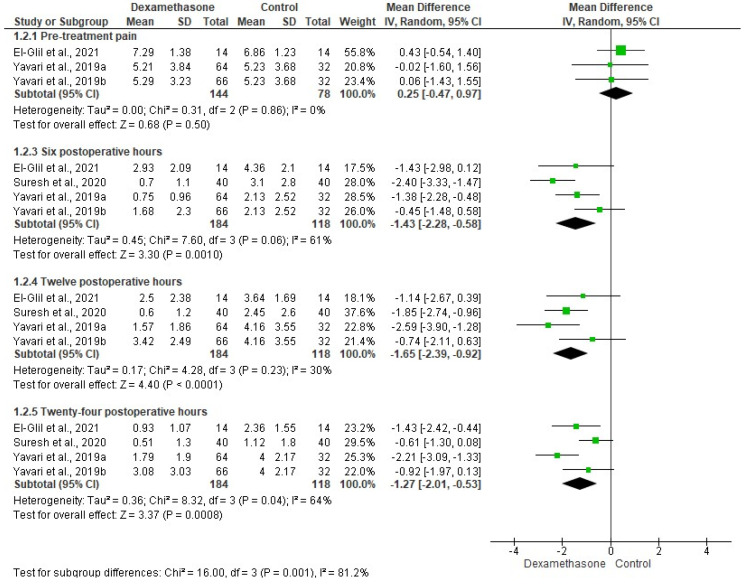
Meta-analysis of the pain intensity, by VAS (*p* < 0.05) [15,22,23].

The pooled evaluation of pain intensity was carried out using the data of three clinical trials (*n* = 302) [15,22,23]. The pre-endodontic pain scores were similar between the dexamethasone group and the control group. In this sense, patients who received dexamethasone had lower pain intensity at 6 (*p* < 0.001), 12 (*p* < 0.0001), and 24 (*p* < 0.0008) postoperative hours when compared to those who were given local anesthetics (Figure 4).

The number of patients who took rescue analgesics in the dexamethasone group was lower in comparison with controls, but a statistical difference was not found (*n* = 58) [10,18]. On the other hand, the risk of adverse reactions was determined with data from five clinical assays (*n* = 436) [9,11,14,20,22]. Only one patient in the dexamethasone group and no patients in the control group presented adverse effects.

### 3.4. The Sensitivity Evaluation and Publication Bias

**Figure 5:**
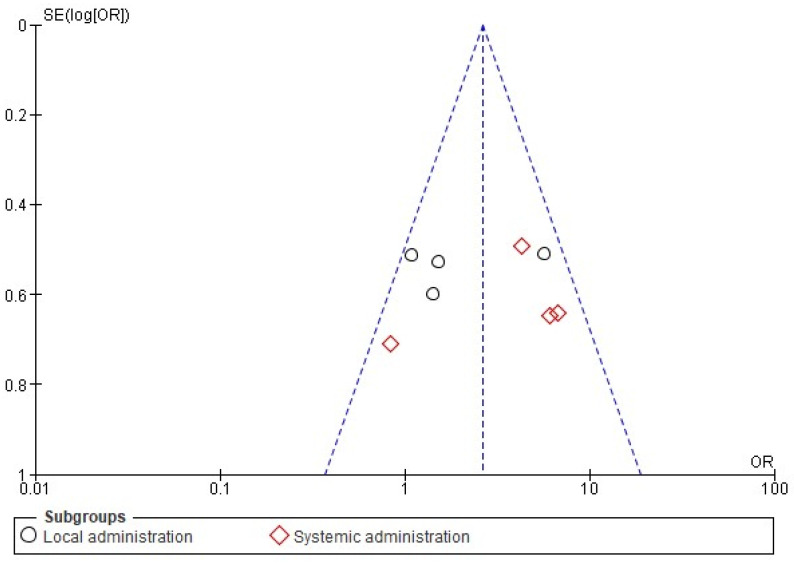
The publication bias [8,9,11,14,16,17,20,21].

The sensitivity analysis did not show important changes in the results of either the anesthetic success index or the pain intensity meta-analyses. Moreover, the visual evaluation identified no publication bias using a funnel plot (Figure 5).

## 4. Discussion

The most important finding of this systematic review and meta-analysis is that systemic—but not local—administration of dexamethasone increased the anesthetic success rate when compared to systemic placebo in patients with SIP. However, the data trend shows a positive effect in favor of the local administration of dexamethasone, for which it is likely that increasing the sample size could achieve this statistical difference. Moreover, dexamethasone produced lower pain intensity scores at 6, 12, and 24 h when compared to the control group. On the other hand, the evaluation of depth and duration of anesthesia, along with the adverse effects of dexamethasone, was not possible, due to the small number of studies reporting these clinical data. No study reported an increase in anesthetic depth, while only one trial reported that dexamethasone prolonged the duration of anesthesia. Aksoy et al. [11] demonstrated that submucosal dexamethasone (349.33 ± 28.24 min) increased the anesthetic effects of IANB using 4% articaine with 1:200000 epinephrine when compared with saline (271.80 ± 17.10 min) [11]. In this context, Pehora et al. [36] carried out a systematic review and meta-analysis, and their findings showed a prolongation of the sensory block when dexamethasone was employed as an adjuvant across a perineural or intravenous route for the peripheral nerve block in upper-limb surgery. Moreover, the local use of the drug could have some advantages in comparison to the systemic administration; for example, the drug is administered exactly where it should exert its therapeutic effect, increasing its concentration and minimizing the time it takes to get to the site where it is needed [37,38,39,40,41,42,43,44].

The Cochrane Collaboration’s risk-of-bias tool showed that all items had a general result indicating a low risk of bias. The items with the highest risk of bias were blinding of patients and medical personnel (performance bias), and blinding of outcome assessment (detection bias) (Figure 2). Moreover, the evaluation of the publication bias of the local administration of dexamethasone showed a slight tendency to publish articles that report an effect in favor of this drug when administered as an adjunct to dental anesthesia in patients with SIP, whereas reports on the systemic administration of dexamethasone showed that published scientific articles present negative results—that is, dexamethasone has no effect on dental anesthesia in patients with SIP. 

Waldron et al. [45] carried out a systematic review and meta-analysis to evaluate the analgesic efficacy of dexamethasone and placebo in patients undergoing general anesthesia. They found better postoperative pain control in patients who received dexamethasone in comparison to those who were given placebo [45]. Knezevic et al. [46] evaluated the effect of perineural dexamethasone added to local anesthesia. The authors reported that dexamethasone reduced postoperative pain when added to the brachial plexus block. However, it increased the anesthesia latency and the duration of the motor block [46]. Nogueira et al. [7] performed a systematic review and meta-analytical assessment of the analgesic efficacy of systemic administration of dexamethasone in patients with SIP. The authors found that systemic administration of dexamethasone was more effective than placebo at 8, 12, and 24 h for pain control in patients with SIP. However, Nogueira et al. [7] did not evaluate the anesthetic success or the adverse effects. Our meta-analysis reports, for the first time, the positive effect of the systemic administration of dexamethasone on the anesthetic success index in patients with SIP.

Some studies have been carried out to determine the adverse effects of dexamethasone in humans. Polderman et al. [47] reported that dexamethasone slightly increases glucose levels, but not the infection risk or wound healing. Moreover, the multi-dose administration of dexamethasone could affect different organs and/or systems, such as the nervous, musculoskeletal, cardiovascular, digestive, endocrine, and renal systems [48]. In this study, dexamethasone was used as a single dose, and the data showed only one clinical trial (one patient in the dexamethasone group) reporting adverse effects. For this reason, pooled analysis was not performed.

The main advantages of this study include an adequate methodology, rigorous and conservative statistical methods, and the available evidence with a lower risk of bias to perform a powerful pooled analysis [29,30,32,33,34,35,49]. Local administration of dexamethasone was performed via the submucosal, supraperiosteal, or intraligamentary routes, and different doses of dexamethasone were used. Therefore, both the routes and the doses could be considered limitations of this study. It is important to note that the results of the local and systemic application of dexamethasone were similar.

## 5. Conclusions

In conclusion, this is the first systematic review and meta-analysis showing that the systemic administration of dexamethasone increases the anesthetic success rate and improves pain management in patients with SIP.

## Figures and Tables

**Table 1:** Included studies.

ID Study and Study Design	Treatments (*n*)	Details of Patients, Dental Procedure, and Evaluation	Important Results/Conclusions
Aggarwal et al., 2011 [8]. Randomized, double-blind, parallel, clinical investigation.	Group A: dexamethasone 4 mg/1 mL (*n* = 23) Group B: 4% articaine and 1:100,000 epinephrine/1.8 mL (*n* = 24). Group C: 4% articaine plus ketorolac 30 mg / 1 mL (*n* = 24). Group D: patients received no treatment (*n* = 23). All treatments were carried out as pre-anesthetic supplemental buccal injection.	ASA I or II patients with pain in a lower molar (moderate-to-severe pain) and diagnosis of SIP with a normal periapical radiograph. Patients without NSAIDs, at least 12 h before the study. All patients were given an IANB using lidocaine 2% and 1:200,000 epinephrine. Success rates were evaluated.	Administration of dexamethasone increased the success rate of local anesthesia.
Aggarwal et al., 2021 [9]. Randomized, double-blind, parallel, clinical assay.	Group A: 1.8 mL of dexamethasone 4 mg/1 mL (*n* = 37). Group B: 1.8 mL of diclofenac from a vial with 75 mg/3 mL (*n* = 38). Group C: normal saline 0.9%/1.8 mL (*n* = 37). All treatments were performed as pre-anesthetic intraligamentary administration.	ASA I or II patients with pain in a lower molar (moderate-to-severe pain) and diagnosis of SIP with a normal periapical radiograph. Patients with prolonged positive response to cold tests. For all patients, IANB was performed using lidocaine 2%, and 1:200,000 epinephrine was employed. The anesthetic success index and the heart rates were assessed.	Intraligamentary dexamethasone administration increased the success rate of anesthesia.
Aksoy and Ege, 2020 [10]. Randomized, double-blind, parallel, clinical trial.	Group A: dexamethasone 8 mg/2 mL (*n* = 30). Group B: tramadol 100 mg/2 mL (*n* = 30). Group C: normal saline 0.9%/2 mL (*n* = 30). All treatments were given (2 mL volume) across the mucobuccal fold of the mandibular molar after anesthesia.	Healthy patients aged 18 to 65 years with a diagnosis of SIP (moderate-to-severe pain) in a mandibular molar, radiographically normal periapical area, and no pain on percussion were included. Patients without analgesic medication, at least 12 h before the study. Patients with prolonged positive response to cold tests. All patients were administered an IANB using 4% articaine with 1:200,000 epinephrine. Postoperative pain intensity, rescue analgesic medication, and adverse effects were evaluated.	Dexamethasone was more effective for pain control when compared with saline.
Aksoy et al., 2021 [11]. Randomized, double-blind, parallel, clinical assay.	Group A: dexamethasone 8 mg/2 mL (*n* = 30). Group B: tramadol 100 mg/2 mL (*n* = 30). Group C: articaine 4%/1.8 mL (*n* = 30). Group D: normal saline 0.9%/2 mL (*n* = 30). All treatments were given (2 mL volume) across the mucobuccal fold of the mandibular molar after anesthesia.	Healthy patients aged 18 to 65 years with a diagnosis of SIP (moderate-to-severe pain) in a mandibular molar, radiographically normal periapical area, and no pain on percussion were included. Patients without analgesic medication, at least 24 h before the study. Patients with prolonged positive response to cold tests. All patients were given an IANB using 4% articaine with 1:200,000 epinephrine. Anesthesia was successful when the pain level of patients included no pain or mild pain. Sensory blockade, duration of anesthesia, success index, and adverse effects were assessed.	Dexamethasone increased the duration of anesthetic activity when compared with saline.
Bidar et al., 2017 [14]. Randomized, double-blind, parallel, clinical investigation.	Group A: dexamethasone 4 mg (*n* = 26). Group B: ibuprofen 400 mg (*n* = 26) Group C: placebo (*n* = 26). All treatments were administered via the oral route.	Patients in good health, over 18 years old, with a lower first or second molar with a diagnosis of SIP (moderate-to-severe pain) were included. Patients with prolonged positive response to cold tests. Patients without analgesic medication, at least 8 h before the study. Standard IANB using 2% lidocaine and 1:80,000 epinephrine was used. Anesthesia was successful when the pain level of patients was no pain or mild pain. The anesthetic success and side effects were evaluated.	Dexamethasone increased the anesthetic success versus placebo.
El-Glil et al., 2021 [15]. Randomized, double-blind, parallel, clinical assay.	Group A: 0.4 mL dexamethasone 8 mg/2 mL (*n* = 14). Group B: 0.4 mL piroxicam 20 mg/mL (*n* = 14). Group C: 0.4 mL mepivacaine 2% and 1:20,000 levonordefrin (*n* = 14). An intraligamentary injection was used for the administration of the drugs.	ASA I or II patients (20 to 60 years old) with pain in a lower molar (moderate-to-severe pain) and diagnosis of SIP with a normal periapical radiograph. Patients without analgesic medication or corticosteroids, at least 24 h before the study. IANB with mepivacaine 2% and 1:20,000 levonordefrin was employed. Post-endodontic pain was evaluated.	Dexamethasone was more effective than mepivacaine/levonordefrin for pain control at 4, 6, 12, 24, and 48 postoperative hours.
Kaushik et al., 2020 [16]. Randomized, double-blind, parallel, clinical trial.	Group A: dexamethasone 4 mg/1 mL (*n* = 34). Group B: distilled water/1 mL (*n* = 35). Treatments were given via the submucosal route.	Patients diagnosed with SIP (moderate-to-severe pain) involving the mandibular molars, without associated pathology. Patients with prolonged positive response to cold tests. IANB 2% lidocaine with 1:200,000 epinephrine was used. Anesthesia success was evaluated.	A similar anesthetic success rate between dexamethasone and distilled water was observed.
Kumar et al., 2021 [17]. Randomized, double-blind, parallel, clinical investigation.	Group A: dexamethasone 0.5 mg (*n* = 21). Group B: ibuprofen 800 mg (*n* = 21). Group C: dexamethasone–ibuprofen combination (*n* = 23). Group D: placebo (*n* = 20). Oral premedication.	ASA I or II patients with pain in a lower molar (moderate-to-severe pain) and diagnosis of SIP with a normal periapical radiograph. Patients having taken no analgesics for at least 12 h before the study. Patients with prolonged positive response to cold tests. For all subjects, IANB was performed using lidocaine 2% and 1:200,000 epinephrine. Overall anesthesia success rate was assessed.	A similar anesthesia rate for dexamethasone and placebo was reported.
Mehrvarzfar et al., 2008 [19]. Randomized, double-blind, parallel, clinical study.	Group A: dexamethasone 8 mg/2 mL (*n* = 50). Group B: placebo (lidocaine 2%/1.8 mL) (*n* = 50). All treatments were given via a supraperiosteal injection (periapical region) after anesthesia.	ASA I or II patients aged 21 to 58 years with a diagnosis of SIP (moderate-to-severe pain) in an incisor or premolar. Patients with positive thermal and electrical tests. The local anesthetic used in all patients was not indicated. Post-endodontic pain was evaluated using VAS.	Dexamethasone was more effective for post-endodontic pain control during the first 24 h than placebo.
Mehrvarzfar et al., 2016 [18]. Randomized, double-blind, parallel, clinical trial.	Group A: 0.2 mL dexamethasone 8 mg/2 mL (*n* = 20). Group B: 0.2 mL lidocaine 2% and 1:80,000 epinephrine mL (*n* = 20). Group C: 0.2 mL saline (*n* = 20). All treatments were administered using a periodontal intraligamentary injection after anesthesia.	ASA I or II patients aged 18 to 65 years with clinical manifestation of SIP (moderate-to-severe pain), without radiographic periapical lesions. Patients with prolonged positive response to cold tests. Intake of opioid analgesics, NSAIDs, corticosteroids, and three-cyclic antidepressants 12 h before treatment. Administration of 1.8 mL of 2% lidocaine with 1:80,000 epinephrine was used to obtain the block the maxillary molars or achieve IANB for the mandibular molars. Post-endodontic pain intensity, the rescue analgesic intake, and adverse effects were recorded.	Dexamethasone reduced the postoperative pain when compared with placebo.
Shahi et al., 2013 [20]. Randomized, double-blind, parallel, clinical assay.	Group A: dexamethasone 0.5 mg (*n* = 55). Group B: ibuprofen 400 mg (*n* = 55). Group C: placebo (*n* = 55). All patients received the experimental the treatments orally.	Good health in patients (aged ≥ 18) with SIP on mandibular first or second molar and a normal periapical radiograph. Patients not taking any analgesics for at least 12 h before the study. Patients with prolonged positive response to cold tests. IANB with 2% lidocaine and 1:80,000 epinephrine was used. The anesthesia success and side effects were assessed.	Dexamethasone increased the anesthetic success in comparison with placebo.
Shokri et al., 2018 [21]. Randomized, double-blind, parallel, clinical investigation.	Group A: dexamethasone 4 mg (*n* = 25). Group B: ibuprofen 400 mg (*n* = 25). Group C: placebo (*n* = 25). All treatments were administered using the oral route.	Emergency patients diagnosed with SIP of a mandibular posterior tooth. IANB was performed. However, the anesthetic agent used was not disclosed. The anesthesia success was analyzed.	Dexamethasone increased the anesthetic success when compared with placebo.
Suresh et al., 2020 [22]. Randomized, double-blind, parallel, clinical study.	Group A: dexamethasone 4 mg (*n* = 40). Group B: piroxicam 20 mg (*n* = 40). Group C: prednisolone 20 mg (*n* = 40). Group D: placebo (*n* = 40). All patients took oral treatments.	Systemic healthy patients aged 18 to 60 years with diagnosed with SIP at a maxillary or mandibular tooth and a normal periapical radiograph. Patients with prolonged positive response to cold tests. Patients having taken no analgesics, steroids, or antibiotics for at least 24 h before the study. Lidocaine 2% and epinephrine 1:100,000 were used to perform the IANB. Post-endodontic pain was assessed at 6, 12, 24, 48, and 72 h.	Dexamethasone reduced the postoperative pain when compared with placebo.
Yavari et al., 2019 [23]. Randomized, double-blind, parallel, clinical trial.	Group A: 0.7 mL dexamethasone 4 mg/mL (*n* = 64) Group B: 0.7 mL long-acting dexamethasone 4 mg/mL (*n* = 66). Group C: 0.7 mL saline 0.9% (*n* = 64). All treatments were administered via the submucosal route after anesthesia.	Healthy individuals aged 20 to 50 years and presenting a diagnosis of symptomatic and asymptomatic irreversible pulpitis with a normal periapical condition. Ibuprofen 1.6 g was used during the two previous days. Patients with prolonged positive response to cold tests. For all subjects, the IANB was performed using lidocaine and 1:100,000 epinephrine. Post-treatment pain was evaluated with the VAS.	Both dexamethasone groups had better postoperative pain relief than saline.

## Data Availability

Data is contained within the article.
